# Production of the antimalarial drug precursor amorphadiene by microbial terpene synthase-like from the moss *Sanionia uncinata*

**DOI:** 10.1007/s00425-024-04558-0

**Published:** 2024-11-20

**Authors:** Hyeonjin Kim, Yelim Lee, Jihyeon Yu, Jong-Yoon Park, Jungeun Lee, Sang-Gyu Kim, Youbong Hyun

**Affiliations:** 1 School of Biological Sciences, Seoul National University, Seoul, 08826 Republic of Korea; 2 Department of Biological Sciences, Korea Advanced Institute for Science and Technology, Daejeon, 34141 Republic of Korea; 3 Division of Life Sciences, Korea Polar Research Institute, Incheon, 21990 Republic of Korea

**Keywords:** Antarctica, Amorpha-4,11-diene, Microbial terpene synthase-likes, Moss, Terpenes, Volatile organic compounds

## Abstract

**Main conclusion:**

The microbial terpene synthase-like of the moss *Sanionia uncinata* displays the convergent evolution of a rare plant metabolite amorpha-4,11-diene synthesis.

**Abstract:**

Despite increasing demand for the exploration of biological resources, the diversity of natural compounds synthesized by organisms inhabiting various climates remains largely unexplored. This study focuses on the moss *Sanionia uncinata*, known as a predominant species within the polar climates of the Antarctic Peninsula, to systematically explore its metabolic profile both in-field and in controlled environments. We here report a diverse array of moss-derived terpene volatiles, including the identification of amorpha-4,11-diene, a rare sesquiterpene compound that is a precursor for antimalarial drugs. Phylogenetic reconstruction and functional validation *in planta* and in vitro identified a moss terpene synthase, *S. uncinata* microbial terpene synthase-like 2 (SuMTPSL2), which is associated with amorpha-4,11-diene production. We demonstrate that expressing SuMTPSL2 in various heterologous systems is sufficient to produce amorpha-4,11-diene. These results highlight the metabolic diversity in Antarctica, but also provide insights into the convergent evolution leading to the synthesis of a rare plant metabolite.

**Supplementary Information:**

The online version contains supplementary material available at 10.1007/s00425-024-04558-0.

## Introduction

All living organisms continuously manufacture thousands of differently structured low molecular weight organic compounds during their life. Of these natural products, terpenes (also known as terpenoids or isoprenoids) constitute the largest and structurally most diverse chemical group, comprising more than 55,000 compounds present in all three domains of life (Connolly and Hill 1991). The majority of terpenes are of plant origin, and terpenes and their derivatives assist the plant species in coping with ever-challenging biotic and abiotic environments (Gershenzon and Dudareva 2007; Tholl 2015).

With increasing evidence for biological functions, the application potentials of terpenes have been also spotlighted. For instance, artemisinin from the annual shrubby plant *Artemisia annua* L. is a sesquiterpene lactone widely used in treating multidrug resistant malaria (Eckstein-Ludwig et al. 2003; Miller and Su 2011). The terpene-derived natural compound is catalyzed from a sesquiterpene olefin precursor amorpha-4,11-diene, and the amorpha-4,11-diene synthase (ADS) of *A. annua* was shown to convert the substrate farnesyl pyrophosphate to amorpha-4,11-diene as its major product (Mercke et al. 2000). By exploiting key enzymes in the biosynthesis pathway, the synthetic production of artemisinin has been thoroughly attempted in genetically engineered *Escherichia coli* and *Saccharomyces cerevisiae* (Martin et al. 2003; Lindahl et al. 2006; Ro et al. 2006; Westfall et al. 2012; Paddon et al. 2013). Similarly, a suite of engineered ADS variants was examined to improve the fidelity of amorpha-4,11-diene production in the synthetic systems (Huang and Fang 2021). However, amorpha-4,11-diene biosynthesis is extremely rare in nature, thereby limiting the route to synthetic manufacture of artemisinin.

The biosynthesis of terpenes is commenced by terpene synthases (TPSs). The enzymes catalyze the formation of basic terpene skeleton by cyclizing isoprenyl pyrophosphate as substrates. Accordingly, mono-, sesqui-, and di-terpene synthases convert geranyl pyrophosphate (C10), farnesyl pyrophosphate (C15), and geranylgeranyl pyrophosphate (C20) to mono- (C10), sesqui- (C15), and di-terpenes (C20), respectively. Reconstruction of TPS lineage diversification in plants further revealed that the plant TPS family can be classified largely into two groups, the plant-type TPSs and the microbial terpene synthase-likes (MTPSLs) (Li et al. 2012; Jia et al. 2016). Plant-type TPSs occur broadly in all land plant lineages, whereas MTPSLs are exclusively detected in non-seed plants, including mosses. Importantly, the members of MTPSLs are more closely related to bacterial and fungal TPSs than plant-type TPSs in their protein and gene structures, suggesting their evolutionary history independent from that of plant-type TPSs (Jia et al. 2018). However, only a small portion of MTPSLs has been studied for their physiological and enzymatic properties while the chemodiversity of terpene biosynthesis has been described in various non-seed plant species (Asakawa et al. 2013; Jia et al. 2018).

This study investigates the biochemistry associated with the production of terpene volatile organic compounds (VOCs) in *Sanionia uncinata* (Hedw.) Loeske, which is a moss species dominating the maritime Antarctica (Ross et al. 1996; Ochyra et al. 2008). Among the cryptogamic vegetation occupying ice-free Antarctic habitats, *S. uncinata* serves as an instrumental niche allowing the Antarctic ecosystem to sustain by accommodating Antarctic small animals and vascular plants (Casanova-Katny and Cavieres 2012; Newsham et al. 2021), assembling endophytic and epiphytic microbial communities (Park et al. 2013; Camara et al. 2021), and being colonized by pathogenic organisms (Tojo et al. 2012; de Menezes et al. 2019). Profiling the headspace volatiles collected from the moss gametophytes inhabiting the Antarctic natural habitats and cultured in the controlled environments, we cataloged a repertoire of terpene VOCs emitted by *S. uncinata*. Notably, the gas chromatography–mass spectrometry (GC–MS) analyses revealed that *S. uncinata* produces amorpha-4,11-diene both in the field and cultivated conditions. Putative *S. uncinata* TPS homologues were predicted, and the enzyme activity assay and heterologous expression of the identified *TPS* homologues revealed one *S. uncinata* microbial terpene synthase-like, SuMTPSL2, as a sesquiterpene synthase that can facilitate the production of amorpha-4,11-diene.

## Materials and methods

### Plant materials and growth conditions

*Sanionia uncinata* (Hedw.) Loeske used in this study was collected in the vicinity of the King Sejong Station on the Barton Peninsula of King George Island. A portion of the moss gametophytes was dissected from the naturally formed beds of *S. uncinata* and then packaged in clean plastic bags for transporting to the laboratory at the Korea Polar Research Institute, Korea. To multiply and establish the experimental strain of *S. uncinata*, the collected gametophytes were cultured on the sterile KNOP media at 22 °C with a 16-h light/8-h dark long day cycle. For the preparation of *S. uncinata* samples that mimic those growing in natural habitats during Antarctic summer, the gametophyte colonies cultured in long day conditions at 22 °C were transferred to a climate chamber that simulates the recorded average temperature and photoperiod of the Antarctic summer. After 6 weeks of culture in the climate chamber, the moss samples were harvested for further studies.

### Phylogenetic reconstruction of terpene synthase family in *S. uncinata*

Terpene synthases from plant, fungal, and bacterial lineages were obtained from InterPro (version 100.0) with conserved catalytic domains of terpene synthases in Pfam (PF01397, PF03936, PF06330, PF19086) and InterPro (IPR050225). Including terpene synthases predicted from *S. uncinata*, total 736 amino acid sequences were analyzed. The sequences were aligned using MAFFT v7.520 (Katoh and Standley 2013) with 1000 iteration of improvement. For maximum likelihood analyses, we used RAxML-NG v1.2.0 (Kozlov et al. 2019) with 1000 bootstrap replications under model (JTT+I+G4). The phylogenetic tree and annotations were visualized using iTOL (Letunic and Bork 2024).

### Profiling terpene volatile compounds

Plant-derived terpene volatiles were collected on polydimethylsiloxane (PDMS) silicon tubing as previously described (Kallenbach et al. 2014). For volatiles trapping from *S. uncinata*, the moss gametophytes were enclosed within polyethylene terephthalate (PET) plastic container in which three PDMS tubes (10 mm long, 1 mm inner diameter, 1.8 mm outer diameter, Carl Roth, Cat. 9555.1) were hanging. PDMS tubes were exposed to the headspace of *S. uncinata* for 24 h in the Antarctic natural habitat and for 20 h in the climate chamber. To capture volatiles from the transgenic *Arabidopsis thaliana* plants that overexpress *S. uncinata* terpene synthases, the fourth or fifth rosette leaf was excised and placed in 8 ml glass vial containing 200 µl ultrapure water. Two PDMS tubes were then exposed to volatiles emitted from the collected leaf for 8 h within the vial. For the transient assays with *Nicotiana benthamiana*, a single agroinfiltrated leaf was excised after 2 days post-infiltration. The prepared *N. benthamiana* leaves were subsequently enclosed in 60 ml glass vial containing 2 ml ultrapure water for 20 h to capture the released volatiles.

To profile the identities of the trapped volatile compounds, thermal desorption GC–MS was conducted using GCMS-QP2020 connected with TD-30 thermal desorption unit (Shimadzu, Kyoto, Japan). The desorbed analytes were injected onto SH-5MS column (30 m × 0.25 mm × 0.25 µm, Shimadzu) together with helium as a carrier gas and then separated by following a series of temperature holds and ramps in oven: (i) 40 °C for 5 min, (ii) thermal gradient ramping to 185 °C (5 °C/min), (iii) ramping to 280 °C (30 °C/min), (iv) holding at final temperature for 0.83 min. The separated compounds were identified by comparing the retention indices and mass spectra with NIST.14 library. C7–C30 saturated alkanes (Supelco) were used to calculate the Kovats retention index. The retention indices were calculated as follows:

$$\text{RI}=100\left(\frac{{t}_{r,t}-{t}_{r,n}}{{t}_{r,n+1}-{t}_{r,t}}+n\right),$$

where *t*_*r,t*_ is the retention time of target compound, *t*_*r,n*_ is the retention time of the alkane standard eluted before the target, *t*_*r,n*+*1*_ is the retention time of the alkane standard eluted after the target, and n is the carbon number of the alkane eluted before the target.

### Analyses of gene expression levels

To estimate the expression patterns of terpene synthase genes in *S. uncinata*, total RNA was extracted from the moss gametophytes grown in the Antarctic habitat and growth chambers using the RNeasy Plant Mini Kit (Qiagen). For cDNA synthesis, 2 μg of total RNA was reverse-transcribed using the M-MLV reverse transcriptase enzyme (Enzynomics) according to the manufacturer’s instructions. Real-time quantification PCR (RT-qPCR) analyses were conducted in StepOnePlus™ Real-Time PCR system (Thermo Fisher Scientific). The housekeeping gene *60S* ribosomal protein L23A was used as a standard for quantification as previously described (Park et al. 2018). Three biological replicates were analyzed for all expression experiments.

To examine transcript abundance of the introduced *SuTPS1* or *SuMTPSL2* genes in the generated transgenic *Arabidopsis thaliana*, total RNA was extracted from rosette leaves using NucleoZol (Macherey–Nagel, Düren, Germany) according to the manufacturer’s protocol. For mRNA expression analyses, 1 μg of total RNA was reverse-transcribed using ReverTraAce qPCR RT Master Mix with gDNA remover kit (Toyobo, Osaka, Japan). The transcript abundance was quantified by RT-qPCR (CFX96, Bio-Rad, Hercules, CA, USA) using the THUNDERBIRD Next SYBR qPCR Mix (Toyobo). The *18S* rRNA in *A. thaliana* was used as a standard for quantification and two or more biological replicates were analyzed for every expression analysis. The sequences of primers used in the expression analyses are presented in Table S2.

### Construction of plasmids, plant transformation, and transient protein expression

To determine the enzymes responsible to produce terpene volatile compounds emitted from *S. uncinata*, the full-length cDNAs of the identified putative terpene synthase genes were cloned by PCR. The cloned cDNAs were then introduced into myc-pBA binary plasmid to drive the moss terpene synthase expression under the control of CaMV *35S* promoter in plants. *A. thaliana* ecotype Columbia (Col-0) was transformed with the constructed *35S::SuTPS1* and *35S::SuMTPSL2* transgenes through *Agrobacterium*-mediated floral dip method (Clough and Bent 1998).

For the transient expression of terpene synthases in *N. benthamiana* leaves, the cDNAs of *SuMTPSL2*, *AaADS* or *SsADS* were introduced downstream of CaMV *35S* promoter in the binary vector pHAtC (Kim et al. 2016). The constructed plasmids were transfected into the leaves of *N. benthamiana* using *Agrobacterium*-mediated infiltration as described previously (Rolland 2018). The sequences of primers used in the plasmid constructions are presented in Table S2.

### In vitro sesquiterpene synthase activity assay

To compare the enzyme activity between SuMTPSL2 and AaADS in vitro, the full-length cDNAs of the terpene synthases were cloned into pET50 protein expression vector. To express the recombinant proteins, the generated constructs were transformed into *E. coli* Rosetta (DE3) strain. Purification of the induced proteins and the subsequent in vitro sesquiterpene synthase activity assay were performed as described previously with minor modifications (Li et al. 2006). Briefly, the transformed *E. coli* cells were cultured in 500 ml LB medium containing 50 µg/ml of kanamycin and 30 µg/ml of chloramphenicol by shaking at 37 °C until the density of bacterial suspensions reached to OD_600_ 0.6. The introduced recombinant proteins were then induced to be expressed in *E. coli* by adding 0.4 mM isopropyl β-d-1-thiogalactopyranoside (IPTG) over 20 h at 15 °C. For protein purification, the bacterial suspensions were pelleted by centrifugation at 5000*g* and 4 °C for 10 min. The pelleted bacterial cells were resuspended with 10 ml Tris-based assay buffer (50 mM Tris–HCl, 15 mM MgCl_2_, 10 mM β-mercaptoethanol, 20% glycerol, pH 8.0) containing 1 mM phenylmethylsulfonyl fluoride (PMSF) and one tablet of cOmplete™ Mini Protease Inhibitor Cocktail (Roche). Soluble protein fractions were separated by centrifugation at 16,200*g* and 4 °C for 40 min, and then purified using Ni-NTA agarose resin (Thermo Fisher Scientific). The size and the amount of purified proteins were confirmed by SDS-PAGE running with bovine serum albumin (BSA, Sigma-Aldrich) serial dilutions as a quantitative standard. The recombinant GFP with His-tag was also purified following the same procedure for a negative control in the enzyme assay.

In vitro sesquiterpene activities of the purified SuMTPSL2, AaADS and GFP proteins were assessed in 100 µl reaction buffer containing 50 mM Tris–HCl (pH 8.0), 15 mM MgCl_2_, 10 mM β-mercaptoethanol, 20% glycerol, 40 µM farnesyl pyrophosphate, and 5 µg of the purified proteins. After incubation at 30 °C for 1 h, the enzyme reaction was terminated by adding 100 µl of hexane, and 2 µl of the eluates with tetralin as an internal standard (IS) were subjected to thermal desorption GC–MS analyses.

## Results

### Terpene volatile production by the moss *S. uncinata in Antarctica*

**Fig. 1:**
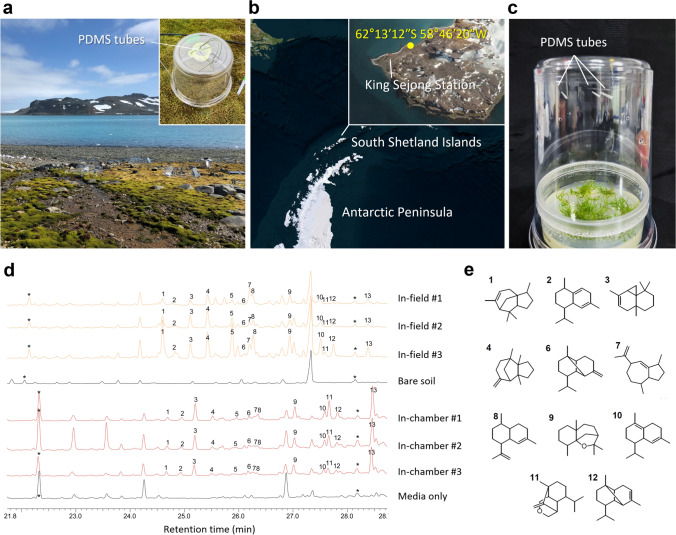
Profiling terpene volatile compounds emitted from the moss *S. uncinata*. **a** In-field capture of headspace terpene volatiles of *S. uncinata*. To collect the moss terpene volatiles, PDMS tubes were exposed for 24 h to the headspace of *S. uncinata* gametophyte bed formed in natural habitat. **b** Experimental site for in-field capture of terpene volatiles from *S. uncinata*. Location of the experimental site is indicated by yellow dot with geographic coordinates on the map. **c** In-chamber capture of headspace terpene volatiles emitted from *S. uncinata*. Terpene volatiles produced by *S. uncinata* were collected by exposing PDMS tubes for 20 h to the headspace of the moss gametophytes. The moss gametophytes were cultured on sterile media in climate chamber. **d** Total ion chromatograms of collected terpene volatiles from *S. uncinata*. Volatiles trapped from bare soil and medium only are presented as negative controls for in-field and in-chamber conditions, respectively. Asterisks indicate saturated alkane internal controls. **e** Putative structures of the identified compounds. The compound numbers correspond to the numbers verifying peaks presented in **d**

To identify the metabolic diversity in Antarctic habitats, we sought to profile terpene VOCs emitted from the Antarctic moss *S. uncinata* (Fig. 1). Headspace terpene volatiles were captured from the moss gametophytes grown under two independent environmental conditions: the in-field Antarctic summer environments (Fig. 1a, b and Fig. S1; Barton Peninsula, King George Island) and the in-chamber sterile culture conditions (Fig. 1c; 22 °C, 16-h light/8-h dark long day cycles). The captured VOCs were then subjected to thermal desorption GC–MS to catalog volatile terpene compounds produced by *S. uncinata* (Kallenbach et al. 2014). The volatile profiling revealed that the moss *S. uncinata* emits sesquiterpene derivatives as its major terpene volatiles (Fig. 1d, e and Table 1), while mono- and di-terpene compounds were hardly detected in our experimental conditions. Although the relative abundance between individual compounds slightly differed, the overall patterns of total ion chromatograms of terpene volatiles under the two examined environments largely overlapped (Fig. 1d). Given that additional biological sources emitting VOCs except for *S. uncinata* were absent under the sterile in-chamber conditions, the sesquiterpenes identified in both experiments do represent the terpene volatiles generated by *S. uncinata* in the Antarctic habitats.

**Table 1:** List of sesquiterpene volatile compounds emitted from *S. uncinata*

No.	RI	Compound Name	Similarity (%)
1	1416	α-Cedrene	94
2	1426	Cadina-1(2),4-diene	90
3	1436	*cis*-Thujopsene	96
4	1448	β-Barbatene	91
5	1465	Unknown	-
6	1473	β-Copaene	88
7	1477	γ-Gurjunene	88
8	1480	Amorpha-4,11-diene^a^	89
9	1506	β-Dihydroagarofuran	91
10	1528	δ-Cadinene	85
11	1532	9-Isopropyl-1-methyl-2-methylene-5-oxatricyclo[5.4.0.0(3,8)]undecane	81
12	1538	α-Copaene	86
13	1564	Unknown	-

Similarity: Mass spectral similarity with reference

^a^The compound was fully identified as amorpha-4,11-diene by comparing mass spectra and retention index with an authentic standard synthesized by recombinant AaADS

Diverse abiotic and biotic factors are known to affect the synthesis and emission of volatiles in plants (Dudareva et al. 2004; Pichersky et al. 2006). Accordingly, there were variations in the relative amount of terpene emissions between the moss grown in natural habitats and those grown in the climate chamber. For instance, the relative proportions of α-cedrene (peak 1), *cis*-thujopsene (peak 3), and β-barbatene (peak 4) in Antarctica differed from those in the climate chamber (Fig. 1d). Similar variation was also observed for 9-isopropyl-1-methyl-2-methylene-5-oxatricyclo[5.4.0.0(3,8)]undecane (peak 11), δ-cadinene (peak 10), and α-copaene (peak 12). Surprisingly, our analyses further elucidated that the Antarctic moss also produces the sesquiterpene artemisinin precursor amorpha-4,11-diene whose biosynthesis has been scarcely demonstrated in nature (Fig. 1d; peak 8). Although amorpha-4,11-diene was found in a minor portion of the profiled sesquiterpenes, its detection under the sterile culture conditions confirms that the rare sesquiterpene natural compound was manufactured by *S. uncinata*.

### Phylogenetic reconstruction of terpene synthase family in *S. uncinata*

**Fig. 2:**
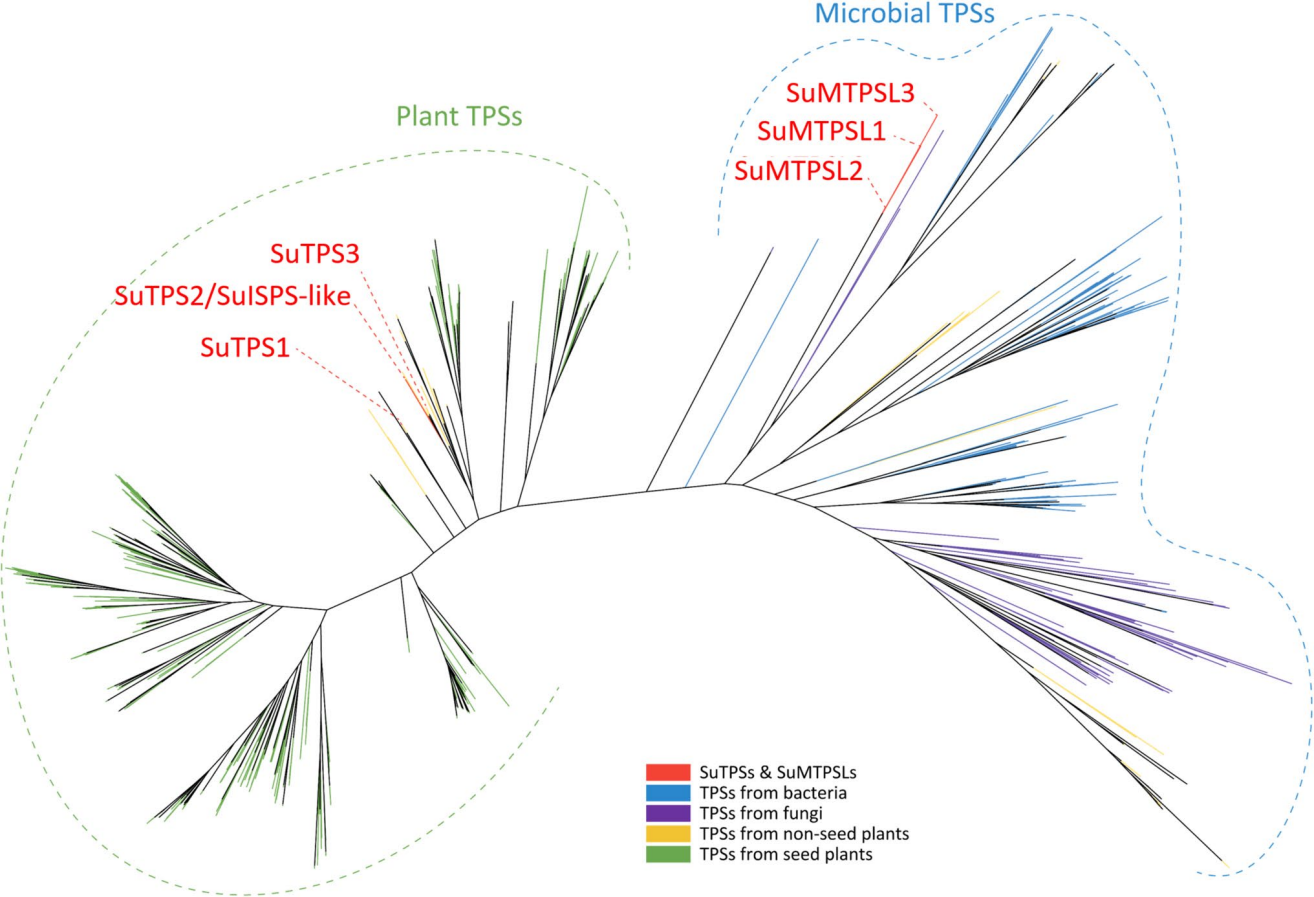
Reconstruction of terpene synthase family in *S. uncinata*. A phylogenetic tree constructed with the predicted terpene synthase candidates in *S. uncinata* is presented. TPSs identified from bacteria (blue), fungi (purple), non-seed plants (orange), and seed plants (green) were included

To characterize the enzyme associated with the observed amorpha-4,11-diene production, we searched for terpene synthases in *S. uncinata*. Using the conserved catalytic domains of terpene synthases (PF01397, PF03936, PF06330, and PF19086) as queries, basic local alignment search tool (BLAST) analyses identified six potential homologous proteins of TPS from the de novo assembled transcriptomes of *S. uncinata* (Li et al. 2022; Yu et al. 2024). The subsequent phylogenetic analyses using 736 terpene synthases classified these putative TPSs into two subgroups (Fig. 2), which we designated *S. uncinata* plant-type TPSs (SuTPSs) and *S. uncinata* microbial terpene synthase-likes (SuMTPSLs). Consistently, three members of SuTPS subclade were grouped together with other plant-type TPSs that are broadly diverged in all land plant lineages (Fig. 2). In contrast, the phylogeny of SuMTPSLs indicated that the group members are more closely related to MTPSLs identified only from non-seed plants. SuMTPSLs also share evolutionary ancestry with TPSs from bacteria and fungi rather than the plant-type TPSs, including SuTPSs (Fig. 2). Therefore, the identified phylogeny of terpene synthase candidates in *S. uncinata* confirms the TPS family structure described in various moss species, retaining both the plant-type TPSs transmitted vertically during land plant divergence and the MTPSLs transferred horizontally from microbial species into non-seed plants (Jia et al. 2016, 2022).

### Molecular characterization of *S. uncinata* terpene synthases

**Fig. 3:**
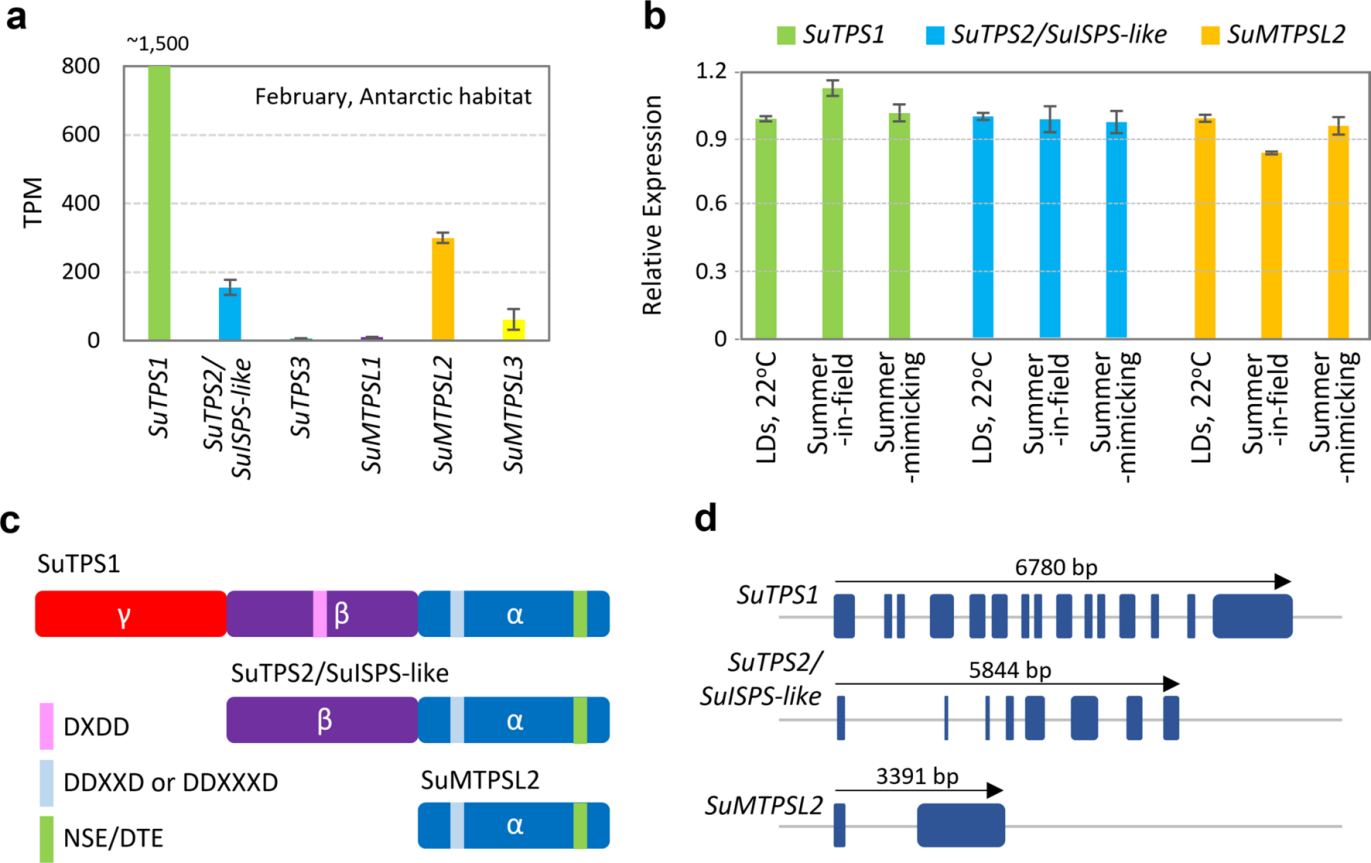
Molecular identification of terpene synthase genes in *S. uncinata*. **a** RNA-seq analyses for normalized transcript abundances of the predicted terpene synthases in *S. uncinata*. Total RNA was extracted from *S. uncinata* grown in Antarctic habitats during summer. TPM: transcripts per million. Error bars indicate standard deviation of three biological replicates. **b** RT-qPCR analyses of *SuTPS1*, *SuTPS2/SuISPS-like*, and *SuMTPSL2* expression. Transcript abundance of each gene was examined using RNA samples extracted from the moss gametophytes grown in constant long day conditions (22 °C), in-field Antarctic natural habitat (summer), and the conditions mimicking Antarctic summer temperature regime, respectively. Error bars indicate standard deviation of three biological replicates. **c** Schematic domain structures of SuTPS1, SuTPS2/SuISPS-like, and SuMTPSL2 proteins. The figure depicts the combination of three modular structural domains, α, β, and γ, occurring in each candidate. The aspartate-rich DXDD (pink) and DDXXD or DDXXXD (light blue), and NSE/DTE (green) catalytic motifs are indicated. **d** Schematics depicting the characterized gene structures of *SuTPS1*, *SuTPS2/SuISPS-like*, and *SuMTPSL2*

To further characterize the TPS candidates at molecular levels, we revisited the de novo assembled transcriptome and characterized the transcript abundances of the predicted *S. uncinata* terpene synthase genes. In the RNA sequencing (RNA-seq) results, two *SuTPSs*, *SuTPS1* and *SuTPS2*, and one *SuMTPSL*, *SuMTPSL2*, exhibited markedly higher expression levels than the remaining *SuTPS3*, *SuMTPSL1*, and *SuMTPSL3* (Fig. 3a). Reflecting the similarities of terpene volatile profiles between the in-field and in-chamber conditions, *SuTPS1*, *SuTPS2*, and *SuMTPSL2* showed similar expression patterns between in-chamber, in-field (summer), and summer-mimicking conditions in the real-time quantitative PCR (RT-qPCR) analyses (Fig. 3b). These suggested that the three abundantly expressed putative terpene synthases may play dominant roles in the production of the detected terpene volatiles, while the possible contributions of the remaining low abundant terpene synthases cannot be excluded.

We then identified the organization of protein and gene structures of *SuTPS1*, *SuTPS2*, and *SuMTPSL2* by cloning the full-length CDS predicted in silico and reinforcing it through mapping RNA-seq reads to the genomic regions. (Fig. 3c, d and Fig. S2). Previous structural comparison of TPSs suggested that the architecture of the TPS protein is modular so that all TPS proteins can consist of one, two or three structural domains, which are now termed as α, β, and γ (Jia et al. 2018). Typical plant diterpene synthases, such as taxadiene synthase from *Taxus brevifolia* (Koksal et al. 2011), are composed of all three domains in an order γ, β, and α, and a similar domain architecture was found in SuTPS1 protein (Fig. 3c). Also, the enzyme had the highest amino acid sequence identity to HpDTC1 (88.64%, Fig. S4a), a bifunctional terpene synthase found in the moss *Calohypnum plumiforme* (formerly *Hypnum plumaeforme*) (Okada et al. 2016).

On the other hand, SuTPS2 showed a didomain structure, consisting of β and α domains with unstructured N-terminal stretch (Fig. 3c and Fig. S3). The deduced polypeptide sequence of SuTPS2 showed 84.59% of identity to that of *C. plumiforme* isoprene synthase (CpISPS) (Kawakami et al. 2023). Moreover, comparison of 3D structure using TM-alignment (Zhang and Skolnick 2005) indicated that both proteins are structurally very similar, with TM-score of 0.87 (Fig. S3). Thus, we could not exclude the possibility that SuTPS2 acts as an ISPS (hereafter referred to as SuTPS2/SuISPS-like), and did not include the enzyme in further investigations.

Supporting the microbial-origin inferred from the phylogeny, SuMTPSL2 displays a single domain structure that adopt only α domain (Fig. 3c), as described in many microbial terpene synthases, such as pentalenene synthase from bacterium *Streptomyces* UC5319 (Lesburg et al. 1997) and trichodiene synthase from fungal species *Fusarium sporotrichioides* (Rynkiewicz et al. 2001). Moreover, similar with most *MTPSL* genes containing no or only one intron, the characterized *SuMTPSL2* gene possessed only one intron, whereas the plant-type *SuTPS1* showed increased structural complexity in the exon and the intron compositions (Fig. 3d). We also confirmed that SuTPS1 and SuMTPSL2 harbor the highly conserved aspartate-rich (DxDD, DDXXD, and DDXXXD) and NSE/DTE catalytic motifs in the domain structure (Fig. 3c and Fig. S4), suggesting their enzymatic functionality.

### Determination of biochemical activities of *S. uncinata* terpene synthases

**Fig. 4:**
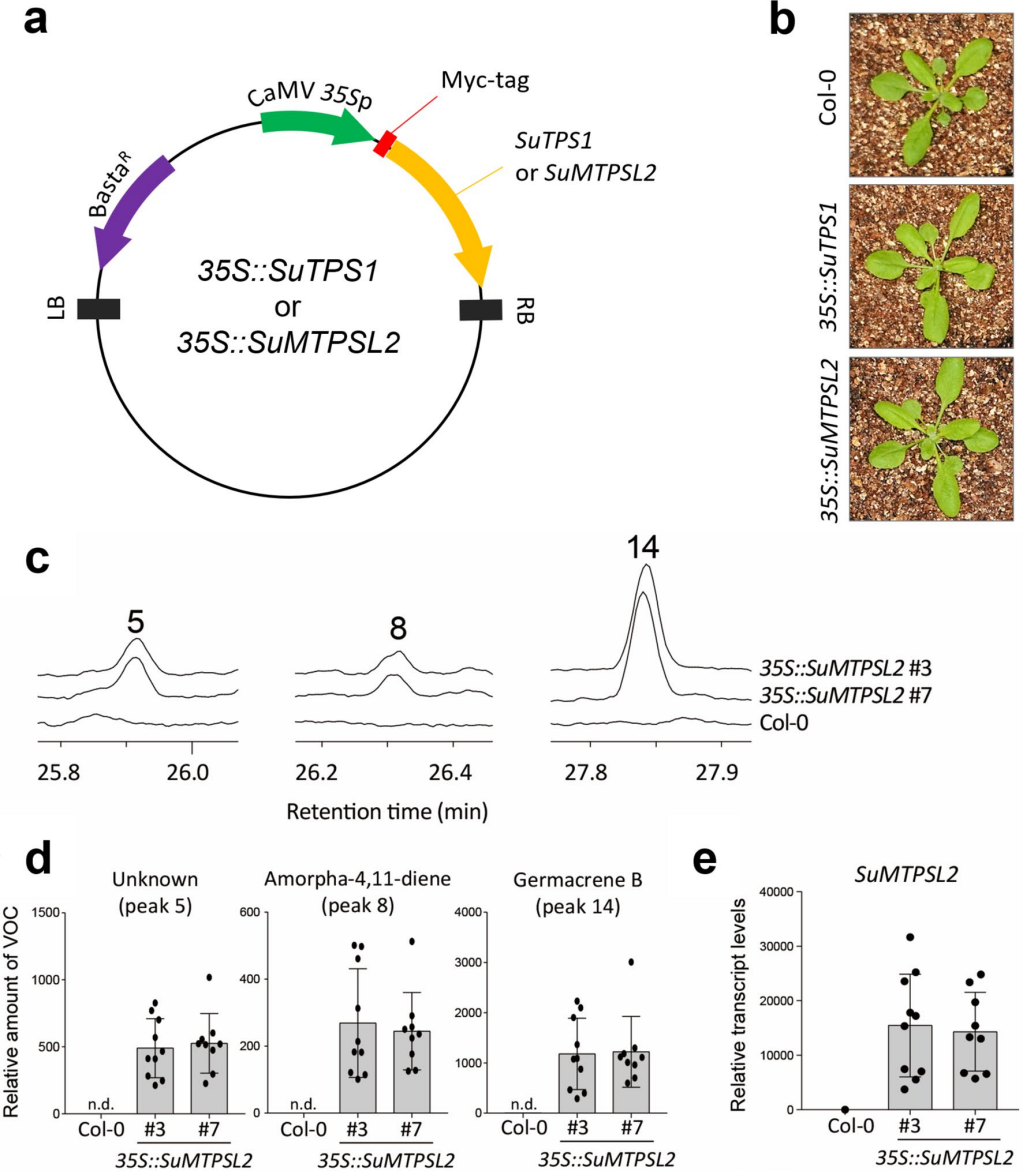
Recapitulation of amorpha-4,11-diene production in *A. thaliana*. **a** Schematics depicting the structure of binary vector harboring *35S::SuTPS1* and *35S::SuMTPSL2* transgenes. **b** Growth patterns of *35S::SuTPS1* and *35S::SuMTPSL2* transgenic plants. Photographs of the representative individual of each genotype are presented. **c** Ion chromatograms of terpene volatiles produced by *35S::SuMTPSL2* plants. To capture terpene volatiles, PDMS tubes were placed with the detached rosette leaf of transgenic or wild-type *A. thaliana* for 8 h in a glass vial. **d** The levels of detected terpene compounds. Relative amounts of volatile compounds presented in c were normalized by leaf area. **e** RT-qPCR analyses of the ectopic expression of *SuMTPSL2* gene in the rosette leaves of *A. thaliana* transgenic plants. Error bars indicate standard deviation of biological replicates

We therefore determined the enzyme activities of SuTPS1 and SuMTPSL2 *in planta*. Since genetic manipulation of *S. uncinata* is yet unavailable, we employed the heterologous expression of the moss terpene synthases in the model flowering plant *Arabidopsis thaliana*. The full-length cDNAs of *S. uncinata* terpene synthases were cloned downstream of Cauliflower Mosaic Virus (CaMV) *35S* promoter in the binary vector myc-pBA (Fig. 4a) (Choi et al. 2011). The constructed *35S::SuTPS1* and *35S::SuMTPSL2* transgenes were subsequently introduced into *A. thaliana* accession Col-0 through *Agrobacterium*-mediated transformation. The ectopic expression of the introduced *SuTPS* family members was confirmed in rosette leaves of the established transgenic plants while the growth and development of transgenic plants did not differ significantly from those of wild-type Col-0 plants (Figs. 4b and S5).

To examine if the introduced *S. uncinata* terpene synthases redirect the metabolic pathways in *A. thaliana*, the headspace collections of terpene VOCs from independent *35S::SuTPS1* and *35S::SuMTPSL2* transgenic plants were profiled. In the model flowering plant, SuMTPSL2 was found to produce germacrene B (Fig. 4c; peak 14) as a major product, with an unknown terpene compound (peak 5), but importantly the GC–MS analyses showed that the introduction of *SuMTPSL2* recapitulates amorpha-4,11-diene accumulation (peak 8). By contrast, the profiles of terpene VOCs were not significantly changed in *35S::SuTPS1* plants (Fig. S5). Thus, the production of amorpha-4,11-diene in *S. uncinata* is potentially catalyzed by the moss terpene synthase SuMTPSL2.

### Amorpha-4,11-diene production by SuMTPSL2

**Fig. 5:**
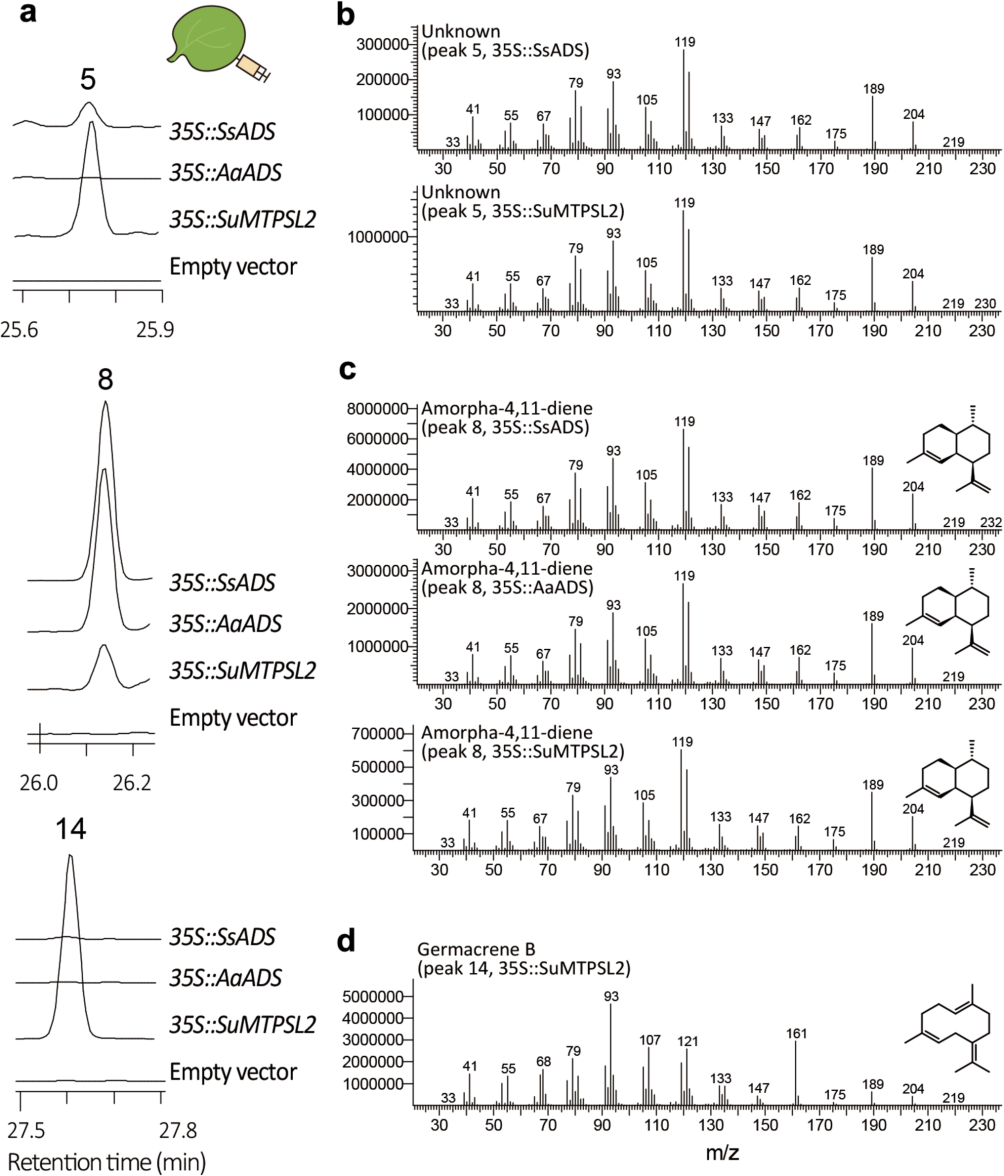
Comparison of enzyme activities between SuMTPSL2 and other amorpha-4,11-diene synthases. **a** Ion chromatograms of headspace terpene volatiles emitted from *N. benthamiana* leaves that transiently overexpress SsADS, AaADS, or SuMTPSL2 enzymes. Terpene volatiles were captured at 2 days post-infiltration. For negative control, the tobacco leaves were infiltrated with empty binary vector used for the transient expression assays. **b–d** Mass spectra of the compounds produced in Agro-infiltrated *N. benthamiana* leaves. Molecular identities of the products were verified by comparing the mass spectra of sesquiterpenes presented in NIST.14 library

The biochemical activities of SuMTPSL2 were further analyzed by comparing with those of previously characterized amorphadiene synthases, *A. annua* amorpha-4,11-diene synthase (AaADS) and *Streptomyces subrutilus* amorpha-4,11-diene synthase (SsADS) (Chhalodia et al. 2023). By independently expressing the enzymes in the leaves of *Nicotiana benthamiana*, we again observed that the transient expression of *SuMTPSL2* facilitated amorpha-4,11-diene production to occur also in tobacco leaves (Fig. 5a, c). However, unlike AaADS that mainly produced amorpha-4,11-diene in the leaves of *N. benthamiana*, the introduced SuMTPSL2 additionally formed the unknown terpene compound (peak 5) and germacrene B (peak 14) (Fig. 5a, b, d). Notably, germacrene B is a compound not profiled in terpene volatiles emitted from the moss *S. uncinata*. This suggests that the sesquiterpene compound might be rapidly processed into other terpene derivatives in *S. uncinata*, while it remains detectable in the heterologous systems possibly due to the absence of associated processing enzymes. Bacterial amorpha-4,11-diene synthase SsADS also produced mainly amorpha-4,11-diene, but minor amounts of the unknown terpene compounds (peak 5) were additionally detected. The mass spectra of amorpha-4,11-diene produced by SuMTPSL2 further confirmed that the molecule is identical to that formed by AaADS and SsADS (Fig. 5c). The unknown compound (peak 5) exhibited the mass fragmentation patterns similar to those of amorpha-4,11-diene (Fig. 5b, c). Thus, the unknown product can be structurally similar with the artemisinin precursor.

The catalytic activity of SuMTPSL2 was also confirmed by in vitro enzyme activity assay (Fig. S6). The purified recombinant SuMTPSL2 converted farnesyl pyrophosphate to the same terpene compounds (peaks 5, 8, and 14) detected in the plants overexpressing *SuMTPSL2*, whereas AaADS produced amorpha-4,11-diene solely. These suggest that SuMTPSL2 may act promiscuously as many terpene synthases do in nature (Pichersky and Raguso 2018; Lanier et al. 2023), thereby conferring terpene diversity in the Antarctic ecosystem. We further noticed that the retention time of amorpha-4,11-diene (peak 8) varied slightly between *in planta* and in vitro enzyme assays, which probably came from instrumental variations. Thus, whether the compounds detected in both experiments are identical was validated by calculating the linear retention index using internal standards included in the assays. The standardization process verified that amorpha-4,11-diene, together with other terpene compounds detected, indeed showed almost identical retention indices among independent profiling analyses (Fig. S7 and Table S1).

Taken together, our combined *in planta* and in vitro biochemical analyses conclude SuMTPSL2 as a sesquiterpene synthase catalytically promiscuous but capable of synthesizing the antimalarial drug precursor amorpha-4,11-diene in diverse heterologous systems.

## Discussion

Among numerous organic metabolites found in all domains of life, terpenes are particularly noticeable given their extreme structural diversity, broad range of biological activities, and the resulted application potentials. To chart such chemodiversity elaborated by the species occupying Antarctica, this study presents a profile of terpene VOCs synthesized by *S. uncinata*, which unexpectedly detected amorpha-4,11-diene production in the moss species (Fig. 1).

In plants, sesquiterpenes often contribute to floral scents that attract pollinators or deter florivores, but these molecules also constitute a crucial part in defense against pathogens and abiotic stresses (Gershenzon and Dudareva 2007). Genetic studies in *Marchantia polymorpha* demonstrated that the liverwort species suppresses herbivory by accumulating mono- and sesqui-terpenes with antifeedant activities in a membrane-bound organelle called oil body (Tanaka et al. 2016; Kanazawa et al. 2020; Romani et al. 2020). Accordingly, the non-seed model plant that genetically impairs the formation of intracellular oil bodies exhibited considerably increased herbivory if exposed to terrestrial isopod *Armadillidium vulgare* (pill bug). These experimental evidences challenge a traditional view that herbivory is less apparent in mosses as they are not sufficient calorie sources. Instead, the results clearly show that many basal land plants also sophisticatedly exploit terpene compounds to defend themselves. In the present study, we conducted the volatile profiling in the growing season of Antarctica during which *S. uncinata* may intimately interact with and host diverse organisms (Tojo et al. 2012; Park et al. 2013; de Menezes et al. 2019; Camara et al. 2021; Newsham et al. 2021). Therefore, the sesquiterpenes produced by *S. uncinata* can serve as mediators of such biological interactions. Supporting these, δ-cadinene (peak 10) detected from *S. uncinata* was shown to possess antibacterial and insecticidal activities in vitro (Perez-Lopez et al. 2011; Govindarajan et al. 2016). Similarly, anti-gnawing activities against small animals and antimicrobial functions of thujopsene (peak 3) were also demonstrated from the oil extracts of coniferous tree *Thujopsis dolabrata* (Ahn et al. 1995; Oh et al. 2011).

Biochemical activities of SuTPS1 and SuTPS2/SuISPS-like remain unclear. We note that their polypeptide sequences and domain structures showed the greatest similarity to those of HpDTC1 and CpISPS from the moss *C. plumiforme,* respectively. HpDTC1 is a bifunctional diterpene cyclase responsible for the conversion of geranylgeranyl diphosphate (GGDP) into syn-pimara-7,15-diene (Okada et al. 2016). Additionally, CpISPS is the first reported isoprene synthase in moss, producing isoprene from dimethylallyl diphosphate (DMAPP) (Kawakami et al. 2023). Based on these, SuTPS1 and SuTPS2/SuISPS-like were also predicted to act as bifunctional diterpene synthase and isoprene synthase, respectively. However, to determine their enzyme activities, further biochemical verification is required.

Amorpha-4,11-diene is a rare natural compound, serving as a precursor for the biosynthetic pathway of antimalarial drug artemisinin. The sesquiterpene synthase AaADS was first characterized to catalyze amorpha-4,11-diene synthesis in the flowering plant *A. annua* (Mercke et al. 2000; Huang and Fang 2021). Recently, new bacterial amorphadiene synthases were identified from *Streptomyces lavendulae* and *Streptomyces subrutilus* (Chhalodia et al. 2023). In this study, our combined *in planta* and in vitro analyses newly identified that *SuMTPSL2* encodes a sesquiterpene synthase, which mediates amorpha-4,11-diene production in *S. uncinata* (Figs. 4, 5). Since MTPSLs do not occur in higher plants (Jia et al. 2018), the synthesis of amorpha-4,11-diene in the moss *S. uncinata* arose independently of that in the flowering plant *A. annua*.

Unlike AaADS that converts the substrate farnesyl pyrophosphate solely to amorpha-4,11-diene, SuMTPSL2 appeared to form multiple products, including germacrene B (Figs. 4, 5 and Fig. S6). The unknown terpene compound (peak 5) with almost similar mass spectra patterns of amorpha-4,11-diene was only produced by SuMTPSL2 and SsADS, but not by AaADS (Fig. 5). Because of the same side product, we suggest that the mechanism of amorpha-4,11-diene synthesis of SuMTPSL2 is much similar with that of bacterial ADS (SsADS) than that of the plant-type ADS (AaADS). Consistently, although the three amorpha-4,11-diene synthases exhibited low similarities in full-length amino acid sequences, the catalytic NSE/DTE motif of SuMTPSL2 (NDVFSYHKE) shares 77.8% sequence identity with that of SsADS (NDVFSLPKE), but only 33.3% identity with that of AaADS (NDLMTHKAE). Moreover, the production of germacrene B suggests that SuMTPSL2 catalyzes initial 1,10-cyclization for germacradienyl cation unlike initial 1,6-cyclization of AaADS (Kim et al. 2006; Picaud et al. 2006).

In sesquiterpene production, germacrenes act as enzyme-bound neutral intermediates that can undergo further reactions to form more products (Rising et al. 2000; Garms et al. 2010; Xu and Dickschat 2023). Co-crystallization of tobacco 5-epi-aristolochene synthase (TEAS) with artificial substrate farnesyl hydroxyphosphonate unraveled a catalytic triad crucial for the processing of the neutral intermediate germacrene A (Starks et al. 1997). Consistently, the directed-mutagenesis using TEAS further demonstrated that the substitution of tyrosine residue in the catalytic triad with phenylalanine (Y520F) led the enzyme variant to release germacrene A as its sole product (Rising et al. 2000). Similar to these observations, a multiproduct sesquiterpene synthase *Medicago truncatula* TPS5 (MtTPS5) forms various sesquiterpenes by employing the reaction sequences that proceed via germacrene intermediate (Garms et al. 2010). The importance of germacrene B (peak 14) has been also discussed thoroughly as a central intermediate in sesquiterpene biosynthesis (Xu and Dickschat 2023), suggesting germacrene B as a potential intermediate in the reaction sequences of SuMTPSL2-mediated terpene production. It would be thus of great interest to determine the biochemistry of SuMTPSL2 and engineer the promiscuity of the moss terpene synthase using the characterized reaction mechanisms for future applications.

## Supplementary Information

Below is the link to the electronic supplementary material.

Supplementary file1 (PDF 2652 KB)

## Data Availability

The data that support the findings of this study are available from the corresponding author upon reasonable request.

## Abbreviations

ADS: Amorpha-4,11-diene synthase
MTPSL: Microbial terpene synthase-like
TPS: Terpene synthase
VOC: Volatile organic compound
