# Supplementary material for: Production of the antimalarial drug precursor amorphadiene by microbial terpene synthase-like from the moss Sanionia uncinata
Source: Planta. 2024 Nov 20;260(6):145. doi: 10.1007/s00425-024-04558-0 (PMC11579073; doi:10.1007/s00425-024-04558-0)
Supplement: Supplementary file 1 — Supplementary file1 (PDF 2652 KB) [file 425_2024_4558_MOESM1_ESM.pdf]

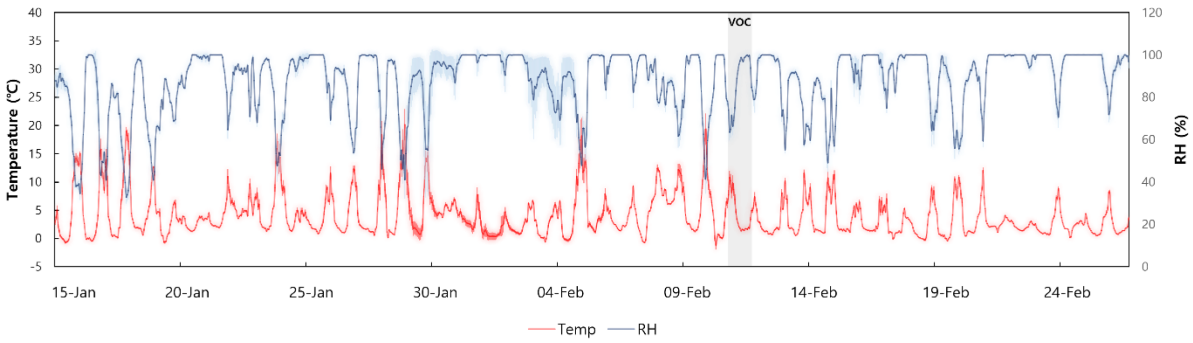

Fig. S1 Temperature and humidity regimes at the Antarctic in-field VOC trapping site. Profiles of soil-surface temperature and relative humidity at the experimental site from 15 January, 2022 to 26 February, 2022 are presented. The in-field VOC capture was conducted on 11 February, 2022 (gray box).

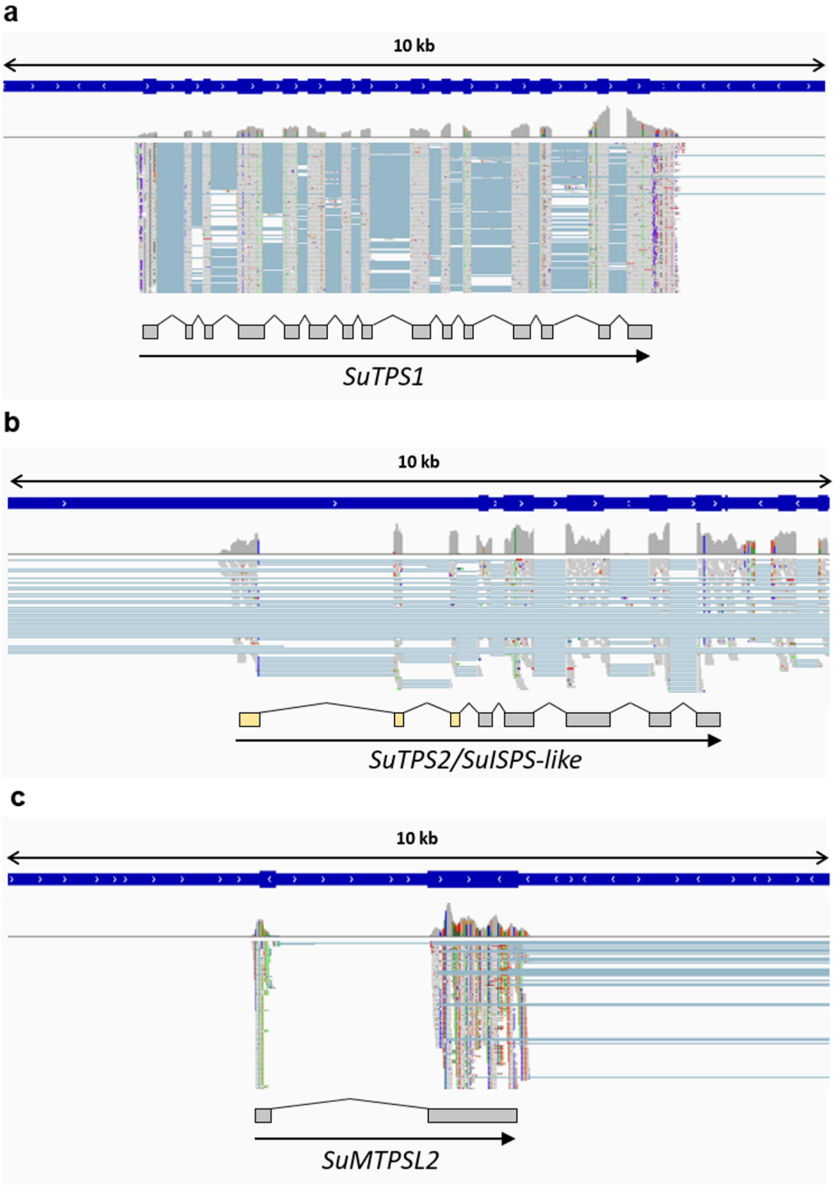

Fig. S2 Characterization of gene structures of *SuTPS1*, *SuTPS2/SuISPS-like* and *SuMTPSL2*. Patterns of RNA-seq read mapping on *SuTPS1* (a), *SuTPS2/SuISPS-like* (b), and *SuMTPSL2* (c) genomic regions are presented. The blue boxes on the blue line at the top are exons annotated *in silico*, while the gray and yellow boxes at the bottom represent exons confirmed by RNA-seq read mapping. Exons annotated only by RNA-seq read mapping are colored in yellow.

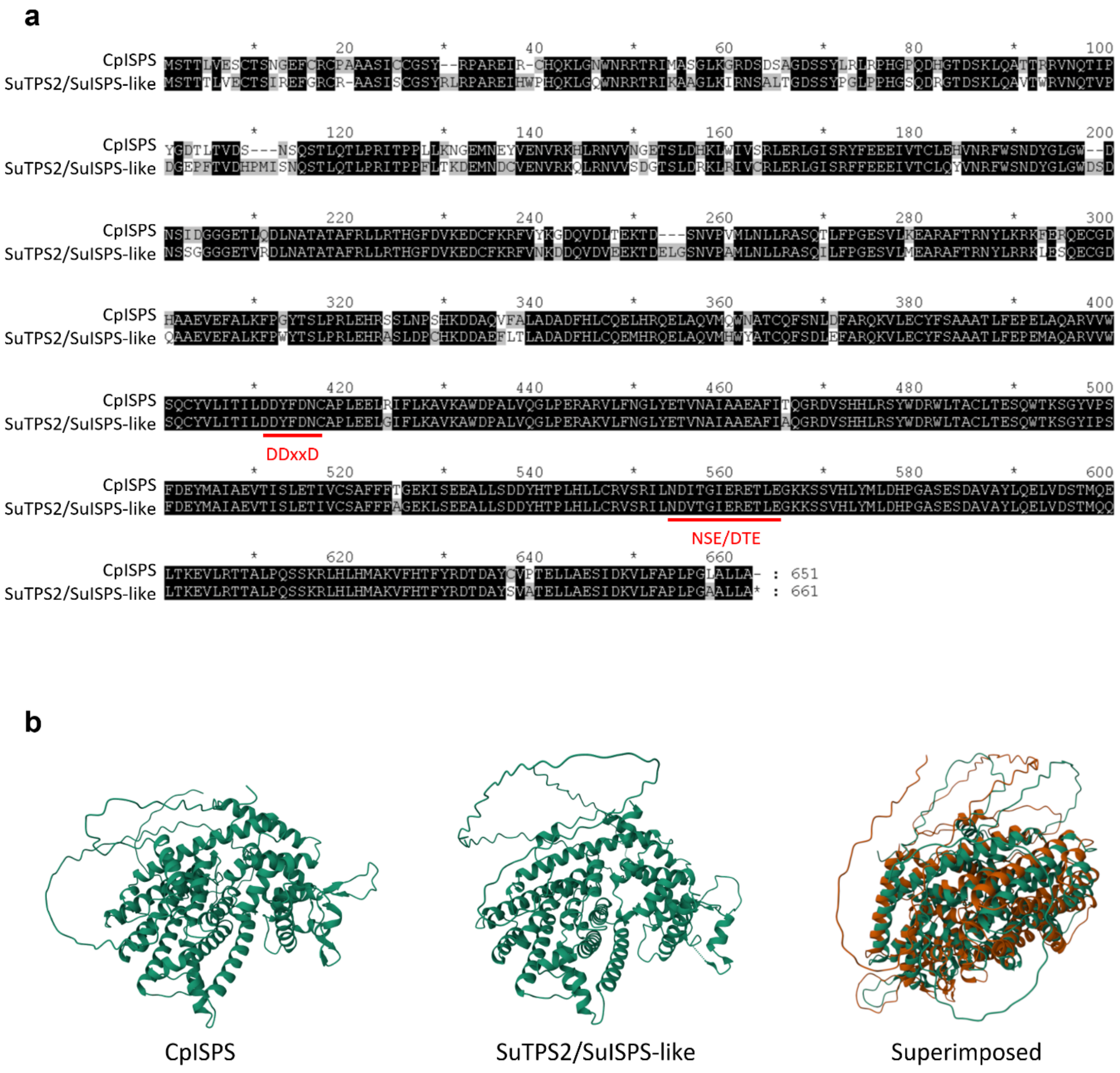

Fig. S3 Sequence and structural similarities between CpISPS and SuTPS2/SuISPS-like. **a** Amino acid sequence alignment of CpISPS and SuTPS2/SuISPS-like. The catalytic DDXXD and NSE/DTE motifs are marked on the alignment result. **b** Predicted three-dimensional protein structures of CpISPS and SuTPS2/SuISPS-like. The protein structures were predicted using the AlphaFold2.

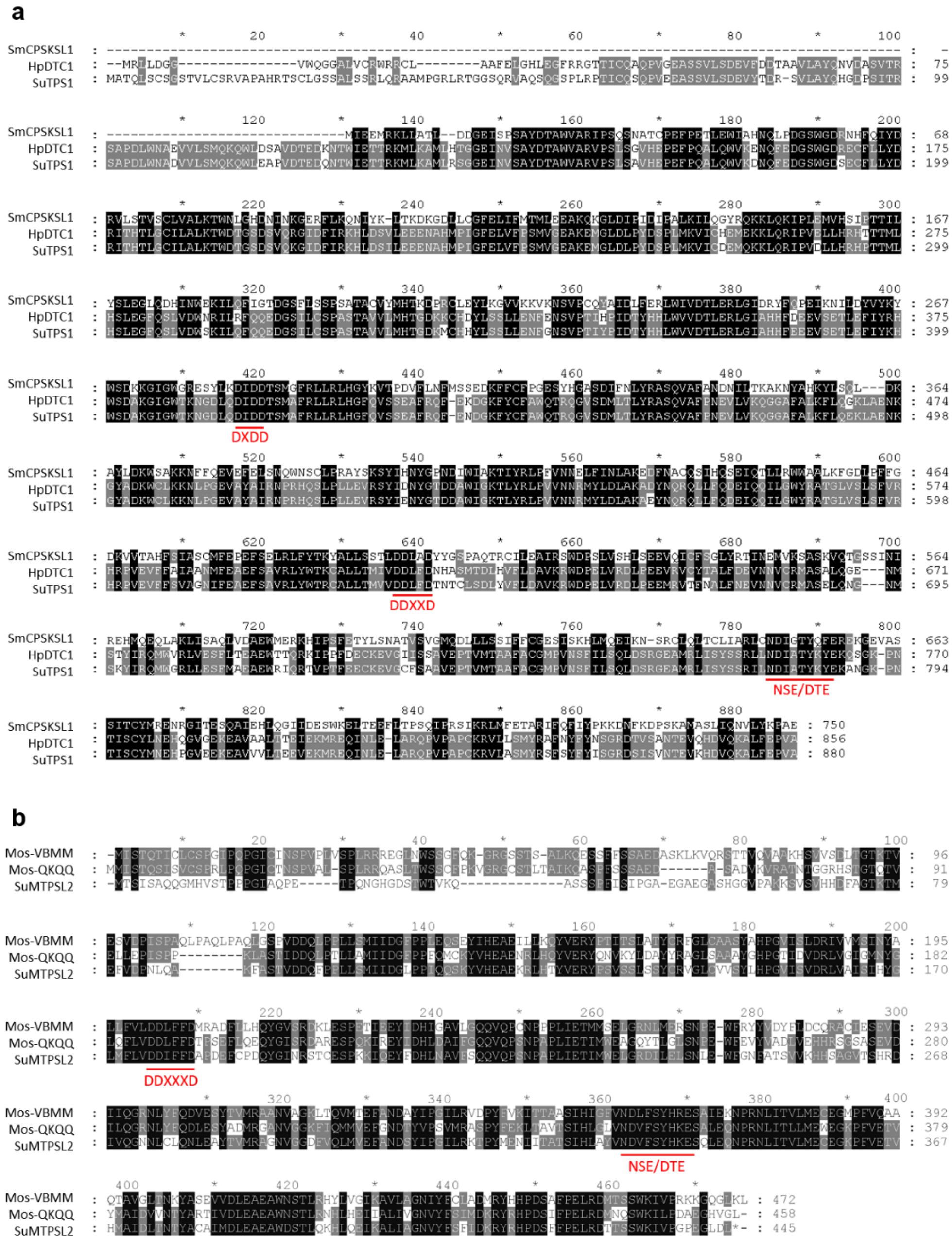

Fig. S4 Conservation of terpene synthase catalytic motifs in SuTPS1 and SuMTPSL2. Multiple sequence alignment results using full-length amino acid sequences of SuTPS1 with plant-type TPSs (a) and SuMTPSL2 with MTPSLs (b) are presented. The catalytic DXDD, DDXXD, DDXXXD, and NSE/DTE motifs are marked on the alignment results. Additional information for the included terpene synthases is presented in Table S3.

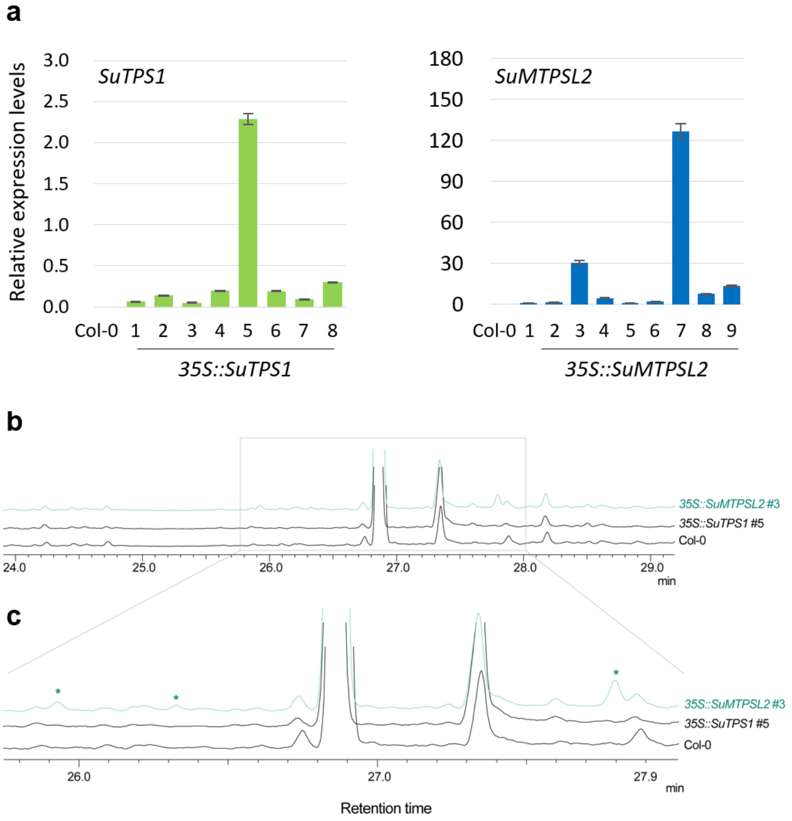

Fig. S5 Introduction of *S. uncinata* terpene synthases into flowering plant *A. thaliana*. **a** RT-qPCR analyses of the ectopic expression of the introduced *S. uncinata* terpene synthase genes in the rosette leaves of *A. thaliana* T1 transgenic plants. Error bars indicate standard deviation of biological replicates. **b** GC-MS analyses of terpene VOCs emitted from rosette leaves of *A. thaliana* T1 transgenic plants that ectopically express *S. uncinata* terpene synthases. Ion chromatograms of terpene volatiles captured from independent transgenic plants are presented. Wild-type Col-0 was included as a negative control. **c** Extracted chromatograms shown in b. Asterisks indicate sesquiterpene compounds specifically induced by the heterologous expression of *SuMTPSL2*.

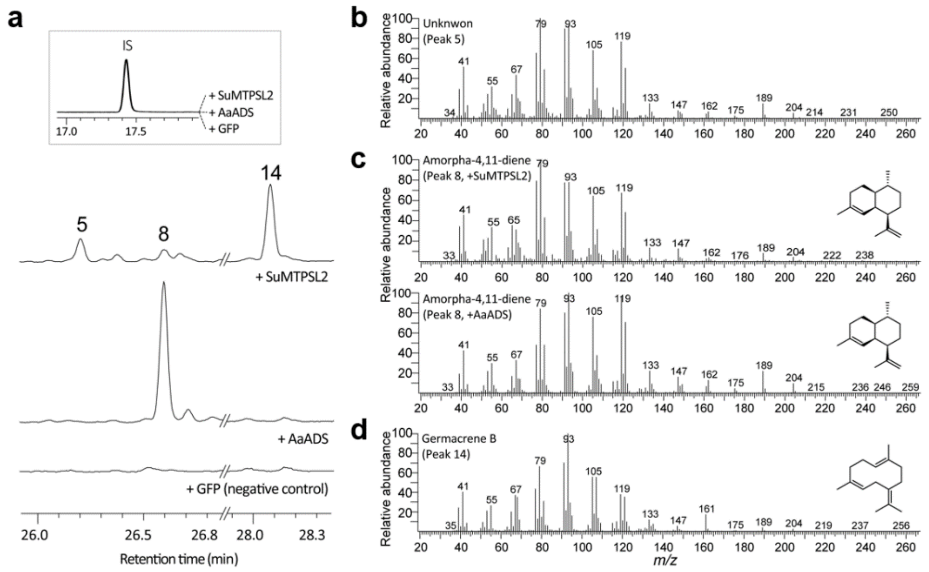

Fig. S6 Comparison of *in vitro* enzyme activities between SuMTPSL2 and AaADS. **a** *In vitro* sesquiterpene synthase activity of recombinant SuMTPSL2 and AaADS. Concentrated products of each enzyme reaction were eluted with hexane containing tetralin (73nM) as an internal standard (IS). Reaction with GFP proteins was included as a non-functional enzyme control. **b-d** Mass spectra of the compounds produced in *in vitro* enzyme assays. Molecular identities of the enzyme products were verified by comparing the mass spectra of sesquiterpenes presented in NIST.14 library.

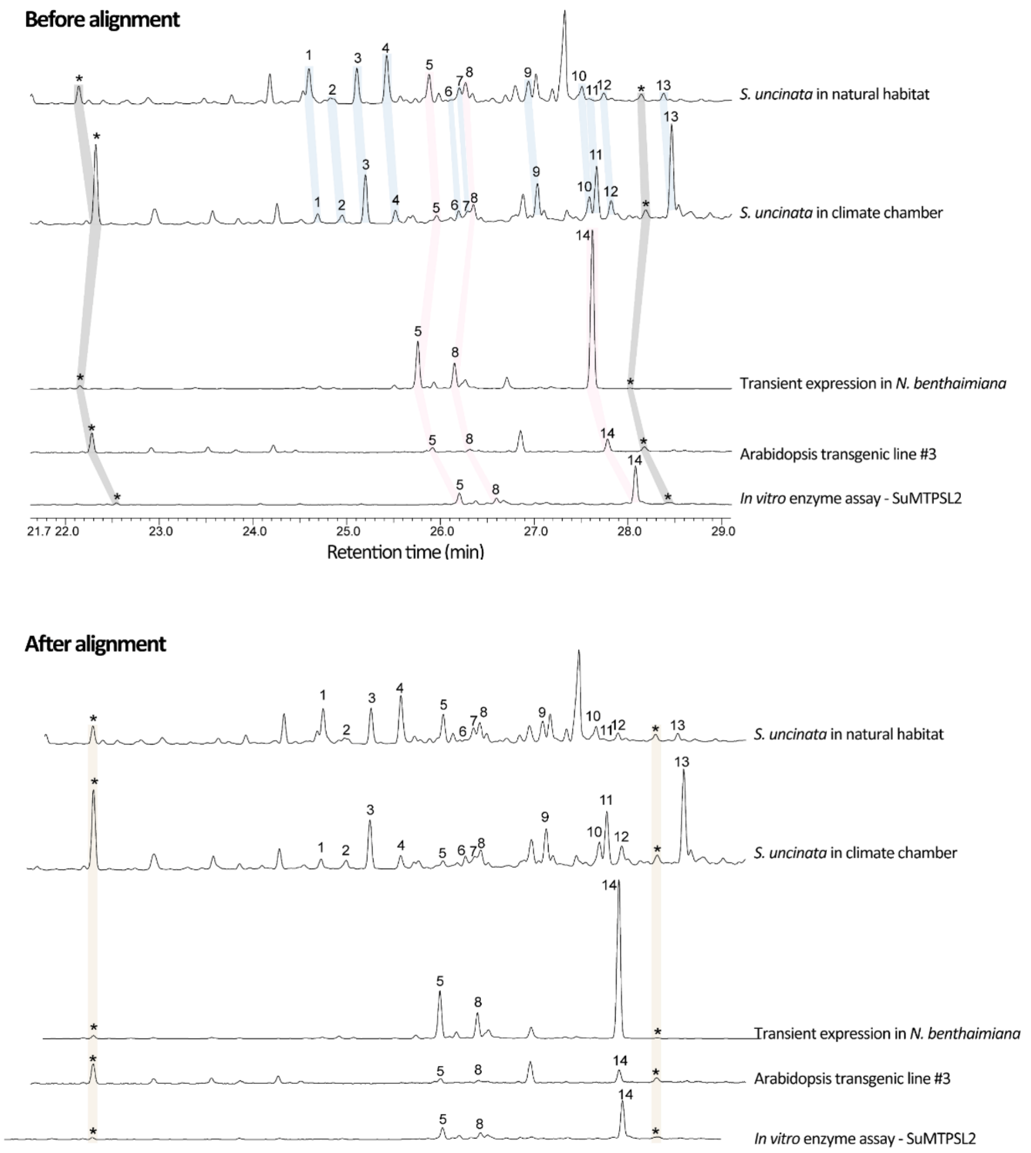

Fig. S7 Standardization of compound profiles using the linear retention indices. Ion chromatograms of terpene volatile profiling from representative experiments are shown at the top. As these experiments were carried out independently, the retention times of the same compounds, including internal standards (asterisks), vary between experiments. To account for these instrumental variations, linear retention indices of target compounds were calculated. The chromatograms standardized with these indices are presented at the bottom. The calculated indices of the detected compounds are presented in Table S1.

Table S1 Linear retention indices of terpene volatiles emitted from *S. uncinata*.

No.	Compound Name	*S. uncinata* in natural habitat		*S. uncinata* in climate chamber		*Arabidopsis* transgenic plant #3		Transient expression in *N. benthamiana*		*In vitro* enzyme assay - SuMTPSL2	
		RT	RI	RT	RI	RT	RI	RT	RI	RT	RI
1	α-Cedrene	24.597	1416	24.691	1416	-	-	-	-	-	-
2	Cadina-1(2),4-diene	24.859	1426	24.953	1426	-	-	-	-	-	-
3	*cis*-Thujopsene	25.108	1435	25.2	1436	-	-	-	-	-	-
4	β-Barbatene	25.426	1447	25.52	1448	-	-	-	-	-	-
5	Unknown	25.878	1465	25.959	1465	25.916	1465	25.758	1464	26.201	1465
6	β-Copaene	26.117	1474	26.194	1474	-	-	-	-	-	-
7	γ-Gurjunene	26.201	1477	26.289	1477	-	-	-	-	-	-
8	Amorpha-4,11-diene	26.268	1479	26.351	1480	26.318	1480	26.149	1479	26.596	1480
9	β-Dihydroagarofuran	26.939	1505	27.035	1506	-	-	-	-	-	-
10	δ-Cadinene	27.509	1528	27.589	1528	-	-	-	-	-	-
11	9-Isopropyl-1-methyl-2-methylene-5-oxatricyclo[5.4.0.0(3,8)]undecane	27.586	1532	27.666	1532	-	-	-	-	-	-
12	α-Copaene	27.743	1538	27.82	1538	-	-	-	-	-	-
13	Unknown	28.38	1564	28.466	1564	-	-	-	-	-	-
14	Germacrene B	-	-	-	-	27.786	1539	27.622	1538	28.08	1539

RT: retention time, RI: retention index

Table S2 List of primers used in the study.

Complementary DNA cloning		
SuTPS1 cDNA	Forward	TTGCATCCGTGAACTTACTG
	Reverse	GCTATGTACTGGCTGCACTG
SuTPS2/SuISPS-like cDNA	Forward	ATGAACGACTGCGTCGAAAATGT
	Reverse	TCACGCCAGTAGAGCCGC
SuMTPSL2 cDNA	Forward	GCAGGAGAGCACACACTCAT
	Reverse	TGCCATAGTCGGCTACACCT
RT-qPCR experiments (in *S. uncinata*)		
SuTPS1	Forward	CGTACGATTCACCGCTAATG
	Reverse	GAGAATGGAGCCATCTTCCT
SuTPS2/SuISPS-like	Forward	AACTGACGAAGGAAGTGCTG
	Reverse	TCACGCCAGTAGAGCCGC
SuMTPSL2	Forward	AATTTTGCCACATCTGTCGT
	Reverse	TACCCGGAATGTAGGAATCA
60S89	Forward	CTAGAGGCTTGGCCTGTTT
	Reverse	TGACAAGGTAGGAAGCAATGA
Transgene construction		
35S::SuTPS1	Forward	GTTCGAAATCGATGGATCATGGCCACTCAGCTCTCATG
	Reverse	CCTAGGCTACGTAGGATCTGCTATGTACTGGCTGCACTG
35S::SuMTPSL2	Forward	GTTCGAAATCGATGGATCATGACGTCGATTTCAGCGCAG
	Reverse	CCTAGGCTACGTAGGATCTGCAGAATTCGCCCTTTGCCATAG
RT-qPCR experiments (in transgenic *A. thaliana*)		
SuTPS1	Forward	CCGTACGATTCACCGCTAATG
	Reverse	GTCGACCAAACTTTGGAACC
SuMTPSL2	Forward	TCTGTCGTCAAACACCACTC
	Reverse	GCAAACTCTACCATCAACTGGAC
18S rRNA	Forward	AAGACGAACAACTGCGAAAGC
	Reverse	AGCAACATCCGCTGATCC
Genotyping PCR of transgenic plants		
35S::SuTPS1	Reverse	GTTGATACGCCAGAACTGATC
35S::SuMTPSL2	Reverse	CGCTGACACTCTTCTTTGC
pro35S-F	Forward	CGCACAATCCCACTATCCTTC

Table S3 List of TPSs and MTPSLs selected for a protein sequence alignment.

Name (Accession number)	Species	Biochemical function	Lineage
SmCPSKSL1 (EFJ14208.1)	*Selaginella moellendorffii*	γ-7,13E-dien-15-ol synthase	Lycophyte
HpDTC1 (BAV01232.1)	*Hypnum plumaeforme* (*Calohypnum plumiforme*)	Syn-pimara-7,15-diene synthase	Moss
Mos-VBMM-MTPSL3 (APB88777.1)	*Anomodon rostratus*	Unknown	Moss
Mos-QKQQ-MTPSL3 (APB88776.1)	*Pseudotaxiphyllum elegans*	Unknown	Moss
